# Seasonal variations of the airborne microbial assemblages of the Seoul subway, South Korea from 16S and ITS gene profiles with chemical analysis

**DOI:** 10.1038/s41598-022-21120-8

**Published:** 2022-11-02

**Authors:** Zohaib Ul Hassan, Hana Cho, Changwoo Park, Yong-Hyeon Yim, Seil Kim

**Affiliations:** 1 Group for Biometrology, Korea Research Institute of Standards and Science (KRISS), Daejeon, 34113 Republic of Korea; 2 Convergent Research Center for Emerging Virus Infection, Korea Research Institute of Chemical Technology (KRICT), Daejeon, 34114 Republic of Korea; 3 Department of Bio-Analytical Science, University of Science and Technology (UST), Daejeon, 34113 Republic of Korea; 4 Inorganic Metrology Group, Division of Chemical and Biological Metrology, Korea Research Institute of Standards and Science, Daejeon, 34113 Korea; 5 Department of Agricultural Biotechnology, Seoul National University (SNU), Seoul, Republic of Korea

**Keywords:** Microbiome, Air microbiology

## Abstract

In this study, we determined the seasonal airborne microbial diversity profiles at SMRT stations by sequencing the 16S rRNA and ITS. Particulate matter samples were collected from air purifiers installed in the platform area of the SMRT subway stations. Three stations that included the most crowded one were selected for the sampling. The sampling was done at each season during 2019. After extracting the total DNA from all seasonal samples, PCR was performed with Illumina overhang adapter primers for the V3–V4 region of the 16S rRNA gene and ITS2 region of the ITS gene. The amplified products were further purified, and sequencing libraries were made. Sequencing was carried with the Illumina Miseq Sequencing system (Illumina, USA) followed by in-depth diversity analyses. The elemental composition of the particulate matter samples collected from the different subway stations were obtained using a WD-XRF spectrometer. The SMRT microbiome showed extensive taxonomic diversity with the most common bacterial genera at the subway stations associated with the skin. Overall, the stations included in this study harbored different phylogenetic communities based on *α- and β*-diversity comparisons. Microbial assemblages also varied depending upon the season in which the samples were taken and the station. Major elements present at the subway stations were from aerosols generated between wheels and brake cushions and between the catenaries and the pantographs. This study shows that the microbial composition of the SMRT subway stations comes from a diverse combination of environmental and human sources, the season and the lifestyle of commuters.

## Introduction

Microorganisms are ubiquitous, not limited to any specific environment or human activities, and are reported to have a prodigious impact on human life^1^. Modern metropolitan residents live in closed environments with prolonged exposure to pollutants. These exposures to pollutants including microbial assemblages of cosmopolitan indoor environments have become significant risk factors to the public health^2^.

In recent years, investigations have been done of the microbiome of urban environments and public mass transit systems, especially subway systems^3,4^. As urbanization progresses, popular demands on mass transportation system, the subway transit networks in major cities are became a major part of regular travelling and the dependency on subways is expected to increase in future worldwide^5^.

Several factors influence the microbiome of the aerosol at subway stations; most important factors are the temperature, humidity and lifestyle of the commuters. However, various other factors are also considered as influential, such as aeration systems and pollution. Several studies investigated the microbial communities of metro systems in highly populous cities including New York City, Boston, Hong Kong, Barcelona, Oslo, Mexico city, Athens and Moscow^4–13^. Seoul, the capital of South Korea, is a densely populated metropolitan area accounting for 12% of the total area of Korea and more than 20% of the total population and is still growing^14^. With an epic increase in the commuters in the Seoul Capital Area (SCA), the subway network is also gaining in reputation and is progressively used as the primary public transportation^15^. In a short period of 40 years, the SMRT has grown into one of the world’s largest public transportation networks with 9 lines with a total length of 327 km (including 290 km underground). On average, 7.2 million passengers per day and 2.6 billion passengers per year use SMRT^16^. No extensive studies have been done evaluating the substances in the SMRT which may be related to the health of the commuters^17^.

A study on personal microbial dispersion reported that individuals can release 10^6^ biological particles per hour and can transmit pathogens to other people and indoor environments^18^. In this context, Seoul commuters on average spend almost 2 h in public transit networks^19^ with an intensive exposure to various particles in indoor environments. Though multiple factors such as different magnitudes of particulate matters, soil and plant debris are involved in the horizontal transfer of biological particles, most dominant factor, the subway network’s architecture, especially the ventilation of aerosol particles, has a dominant effect on complex subway environments^20,21^. In this study, an annual investigation of the SMRT microbiome in 2019 was done with next generation sequencing of 16S rRNA and ITS2 genes and chemical analysis of the aerosols with a wavelength dispersive X-ray fluorescence (WD-XRF) spectrometer. Particulate matter samples were collected from air purifiers installed in the platform area of the SMRT subway stations. Three stations including the most crowded one were selected for the sampling. The average daily passengers of station A, B, and C were about 76, 200 and 52 K, respectively. The microbiome of the SMRT was also compared with other urban microbiomes of the world, showing the unique and common features of the SMRT microbiome.

## Results

### Bacterial phylum at the different subway stations

We have studied 3 major SMRT subway stations and analyzed the 16 samples collected during the different months of year 2019 for this study. All samples received sufficient coverage (> 20,000 reads after filtering using EZbiocloud). The reads were normalized to 20,000 reads for each sample. Total 13,202 OTUs were recovered. The most abundant bacterial phyla were *Actinobacteria, Proteobacteria, Bacteriodetes, Firmicutes, Cyanobacteria, Deinococcus-Thermus* and *Choloroflexi* (Fig S1). *Actinobacteria* with a relative abundance of 39.6% was the most dominant phylum observed at all three stations, followed by *Proteobacteria* (33.3%), *Firmicutes* (11.4%), *Cyanobacteria* (4.9%), *Deinococcus-Thermus* (4%), *Bacteroidetes* (3.9%) and *Chloroflexi* (0.61%). *Cyanobacteria* were detected in higher relative abundance at subway station B and C. *Chloroflexi* was observed at station C while absent at the other stations.

### Characterization of the bacterial community at the genus level

Skin-associated bacterial genera with an average abundance including *Cutibacterium* (4.7%), *Micrococcus* (3%), *Staphylococcus* (3.3%), *Enhydrobacter* (3%), *Corynebacterium* (2.7%) were detected in the aerosols of the SMRT stations. C*utibacterium* (previously known as *Propionibacterium)* is a nonsporulating, gram-positive anaerobic bacillus that is regarded as commensal bacteria on the skin^22^. However, an exception was observed in the Athens subway stations where *Paracoccus* was the most abundant genus in the air with a mean value of (8.0%)^9^ while the SMRT stations had a mean abundance of *Paracoccus* (2%). Moreover, some other significant skin-associated bacterial genera were also present at the SMRT Stations including *Kocuria* (4%), *Deinococcus* (3.7%), *Methylobacterium* (2.7%), and *Rubellimicrobium* (2.3%) (Supplementary Table S2). In addition, the air-associated genus *Sphingmonas* (3.7%), the fresh-soil-dwelling *Blastococcus* (4%) and *Staphylococcus* (3.3%) were the most common genera in the SMRT stations. Although broad-spectrum inclinations are described, distinctive and unique bacterial communities could be observed for some of the samples (Fig S4) similar to interpretations reported by some studies^5,12^.

### Seasonal variations in the bacterial compositions at the SMRT stations

**Figure 1:**
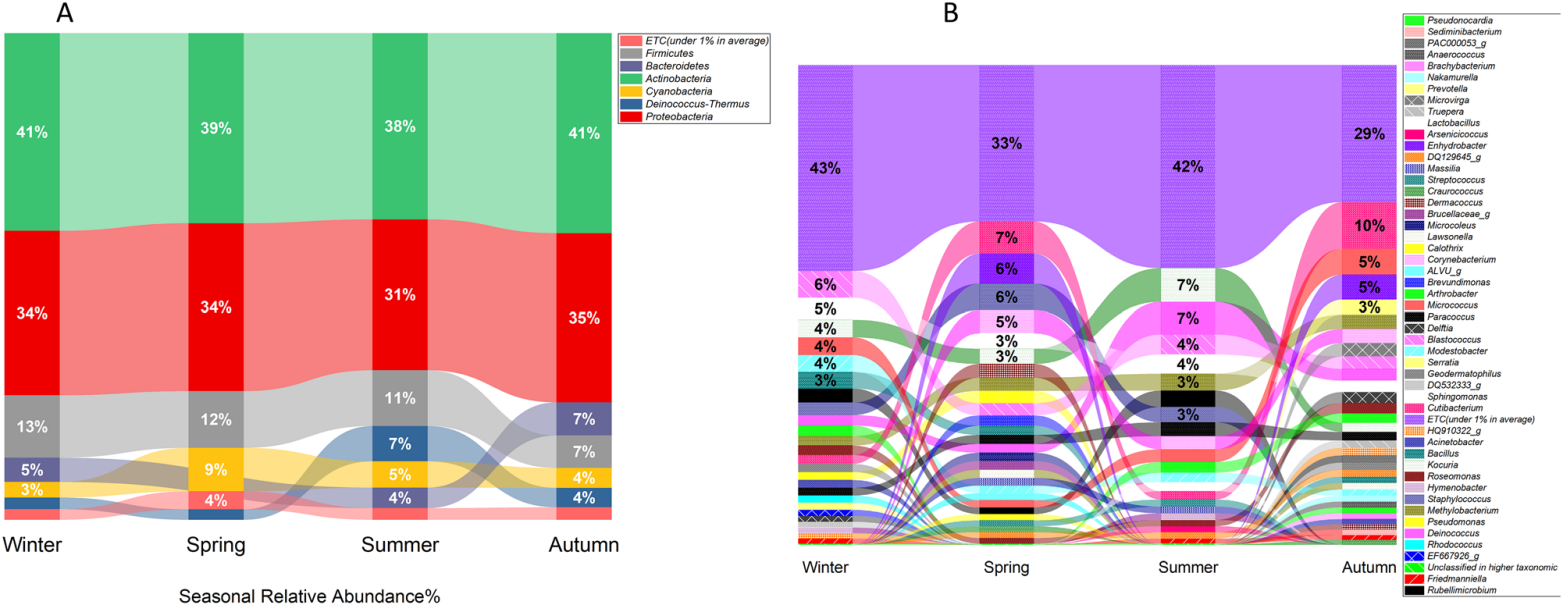
Seasonal bacterial composition at SMRT Stations; (**A**) Major phyla detected during the different seasons (Winter, Spring, Summer and Autumn). (**B**) Most abundant bacterial genera identified at SMRT stations during different seasons.

To analyze the seasonal fluctuations in the bacterial communities, we classified the samples into four seasonal groups: Winter, Spring, Summer, Autumn. Abundance plots of the phyla across the seasons showed a comparatively constant distribution (Fig. 1); however, *Cyanobacteria was* observed to be more abundant during the spring and summer which was in contrast to *Cyanobacteria* being more prevalent in the summer and autumn at the Oslo, Norway subway^11^.

*Cyanobacteria* are a phylum of phototrophic bacteria. The capability to carry out oxygenic photosynthesis (water-oxidizing, oxygen-evolving and plant-like photosynthesis) is the most prominent feature of this phylum^23^. *Firmicutes* showed similar seasonal drifts with the Oslo subway. Compared to the season specific microbiome studies of subways on a family level^11^, *Micrococcaceae* existed as the major bacterial family, showing simila r prevalence trends for the Oslo subway (Supplementary Table S3). *Micrococcaceae* are non-motile and non-spore forming bacteria which include aerobic and facultative anaerobic organisms diversely spread throughout nature and a part of the flora of the human skin^24^. Some abundant bacterial families were found at the Oslo subway but were absent at the SMRT and vice versa (Supplementary Table S3).

A few genera showed the seasonal patterns; for example, *Kocuria* was strongly related to the summer season with an abundance of 7.1% while *Bacillus*, *Sphingomonas* exhibited a seasonal pattern during the winter and *Cutibacterium* was abundant during autumn and spring. The remaining genera showed no significant seasonal variations.

### Bacterial diversity analysis

**Figure 2:**
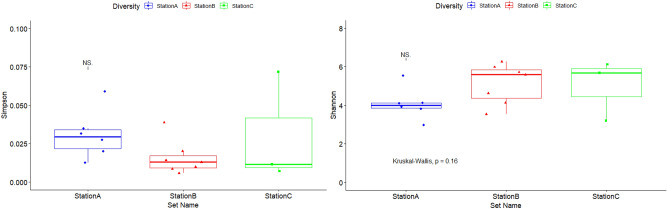
*α-*diversity indices between the 3 SMRT stations. Shannon and Simpson (Wilcoxon rank-sum test *p* < 0.05).

All *α-*diversity indices showed constant results for certain comparisons (Supplementary Table S5). Collectively, SMRT station B had a higher taxonomic diversity than the other SMRT stations in this study (Fig. 2).

**Figure 3:**
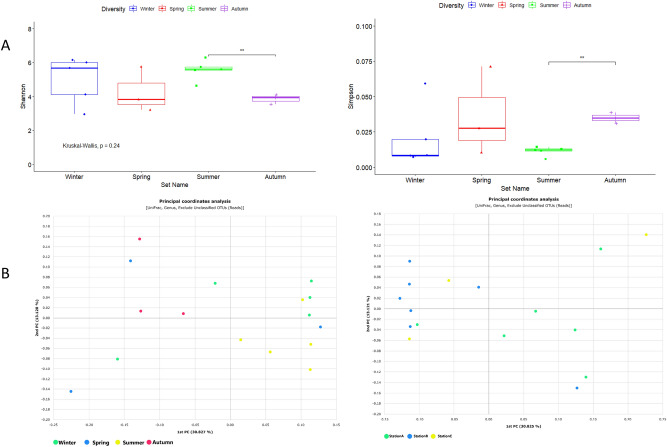
(**A**) *α-*diversity indices of seasons; Shannon and Simpson Indices showed a significant difference of airborne bacterial communities at SMRT stations during summer and autumn. (**B**) Principal Coordinate Analysis (PCoA) between SMRT stations and seasons. PCoA plots of phylogenetic dissimilarities between samples of different SMRT stations were constructed on UniFrac distance metric. A significant difference was observed between SMRT stations B and A also in the summer and autumn seasons.

The comparisons were made by a raw contrast of the diversity indices found in other subways. The peak of the Shannon diversity index (H′ = 5.5) was observed during the summer season. A few previous studies have also specified a relatively greater abundance of airborne microbiomes during the summer season which is probably due to the rise in temperature^25–28^ and high wind speed that can migrate the microbes^29^. All alpha diversity estimators *showed a contrary outcome with a higher diversity in the summer at the SMRT compared to another indoor environment study with more diversity in the spring and fall*^30^*.* A significant diversity difference was recognized between the summer diversity with the highest diversity indices and the autumn with the lowest ones (Fig. 3).

### Major fungal taxa present in the SMRT subway stations

We have studied 3 major SMRT subway stations and analyzed the 16 samples collected during the different months of year 2019 for this study. All samples received sufficient coverage (> 10,000 reads after filtering using EZbiocloud). The reads were normalized to 10,000 reads for each sample. The aerosol fungal diversity at Seoul subway stations was mainly attributed to *Ascomycota* and *Basidiomycota* which was previously observed in a metro station study^9^ and an outdoor study^31^. In our study, within the fungal ITS libraries, the overall most abundant taxa belonged to *Ascomycota* (Mean = 56.11%) followed by *Basidiomycota* (Mean = 19.14%) similar to the findings in the Athens metro study^9^. Remarkably, the third most abundant taxa in the SMRT stations were classified as plants (Mean = 13.91%). These plant taxa may have originated from local centers of diversity or consumption by local population on a regular basis^32^. Similar interesting findings have also been reported in the Mexico metro and in an urban center in Denver^4,28^. The composition and diversity of an aerobiome are strongly linked to the coverage of plantation within a given radius of a site^33^. Other major fungal phyla were soil-associated *Mucoromycota* (2.1%), a potential candidate for biodiesel production^34^ and an unclassified Fungal phylum (Fungi_p) with an average abundance of 5.3% observed during the study.

Overall, 15 fungal classes were detected across all stations with *Eurotiomycetes* having the highest abundance (Mean = 27.4%) compared to the Athens metro (4.3%)^9^. Other major classes (mean abundance) at the SMRT stations were as follows: *Dothideomycetes* (14.3%), *Agaricomycetes* (13.1%), *Saccharomycetes* (9.3%), and *Sordariomycetes* (6.7%). A composition difference in the health-related fungi was observed between the SMRT and Athens metro. The most dominant fungal genus detected in this study was *Aspergillus* (21.9%) which can cause allergic reactions and lung infections called Aspergillosis^35,36^. *Candida* (8.3%) is a skin inhabited genus and can cause severe skin infections^37^. The unclassified genus of *Capnodiales* (6.5%) was also identified at New York subway platforms^12^; *Capnoidales* can cause Chromoblasomycosis-like infection in the human skin^38^. *Nigrospora* (2.6%) also was reported to infect the skin in HIV patients^39^. This outcome suggests that the dominant taxa of the SMRT stations belonged to the skin flora.

### Seasonal variation in the fungal composition

**Figure 4:**
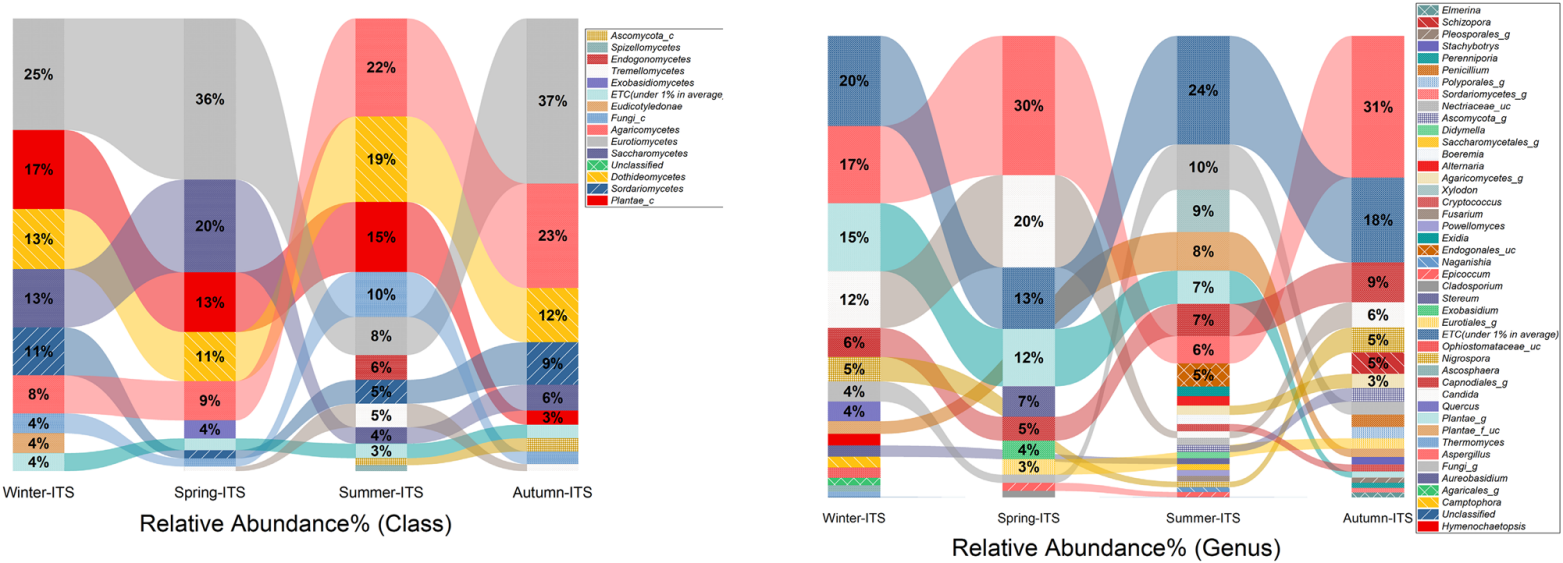
Seasonal Composition of major fungal classes and genera.

*Ascomycota* was the most abundant phylum throughout all the seasons. It was most abundant during spring (69.7%), autumn (66.5%) and winter (62.6%) and the lowest during summer (39.3%). These results are not similar to the indoor study of swine houses in South Korea^40^ where *Ascomycota* was dominant in the summer. The low relative abundance of *Ascomycota* in the summer may linked with the unclassified Fungal phylum (unclassified Fungi_p) occurrence raised from spring (1.7%) to summer (10%). The prevalence of the unclassified Fungal phylum was also reported in high amounts in a large drinking water source during the summer season^41^. *Basidiomycota* was the most dominant in summer (28.2%) and autumn (26.5%) while its abundance was low in winter (10.3%) and spring (14.2%). Interesting findings were drawn by Plantae_p (plant derived), which were in rich abundance during all three seasons except for autumn when the relative abundance decreased to 3%. This finding might be related to more blooms and greenery during the other months. *Mucoromycota* was found only in the summer with a relative abundance of 5.5% showing a strong seasonal trend. *Mucoromycota* recently was found as a causal agent of eye diseases (Mucormycosis) in COVID19 patients in India^42^. The emergence of *Mucoromycetes* infections may affect the public health in the near future. Most dominant class throughout the samples was *Eurotiomycetes*. It was highly abundant during all three seasons except in summer when it decreased significantly from the highest in autumn (36.5%) to the lowest in summer (8.3%). This result might be due to the abundance of *Dothideomycetes* that increased in the summer up to 18.8% from winter (13.2%), autumn and spring (11% each). *Agaricomycetes* exhibited large proportions in the first half of the year in the autumn and summer seasons (23% and 21%), while it decreased in the winter and spring (8% each) (Fig. 4). All these classes were also reported in a previous indoor environment study of swine houses in South Korea^40^, but the dominant seasons were different.

The most abundant genus *Aspergillus* showed a relatively high abundance in spring and autumn (30%) while it remained on the low side during winter (16.8%) and minimal in summer (5.9%). *Candida* was the second most abundant genus with maximum abundance in the spring (20%), decreasing in the winter (12.2%) and autumn (5.5%) and the lowest in the summer (1.5%). The decrease of *Aspergillus* and *Candida* in the summer might be due to some genera having a strong relationship to high temperatures, for example, unclassified *Capnodiales_g,* Unclassified *Endogonales_g* and *Xylodon* were only present in the summer. However, an increase in the plant-derived taxa may also influence the proportions of other genera in the summer season (Fig. 4). Remarkably, traces of *Quercus* (Oak) (4.3%), one of the most common evergreen trees in South Korea and a dominant member of the Korean peninsula evergreen forests^43^, was also identified in the winter.

### Fungal diversity

**Figure 5:**
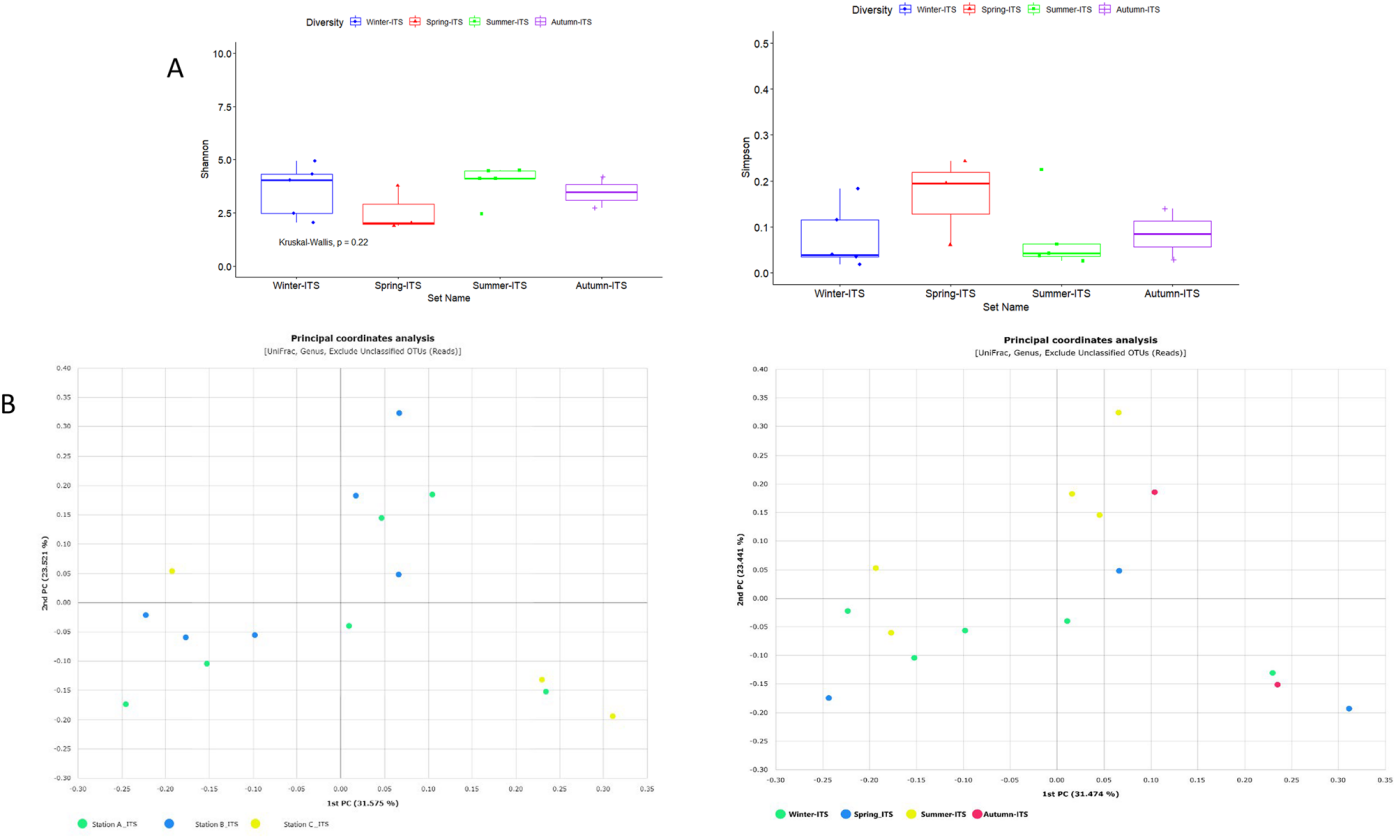
(**A**) *α-*diversity indices of seasons; Shannon and Simpson Indices, no significant difference was observed during the seasons. (**B**) Principal Coordinate Analysis (PCoA) between SMRT stations and seasons.

Species richness estimators suggest SMRT station B had the highest richness among all three stations (Supplementary Table S7). The significant difference of species richness between station B and station A can be seen in Fig. S8. However, the diversity indices mirrored no significant difference between any of the stations shown in Fig. S8. The Shannon diversity index was lower than the weekday study of the Athens metro^9^. Although the diversity indices were highest in summer, no significant difference was observed in the seasonal fungal diversity Fig. 5*.*

### Comparison between the SMRT stations and outdoor micorbiomes

**Figure 6:**
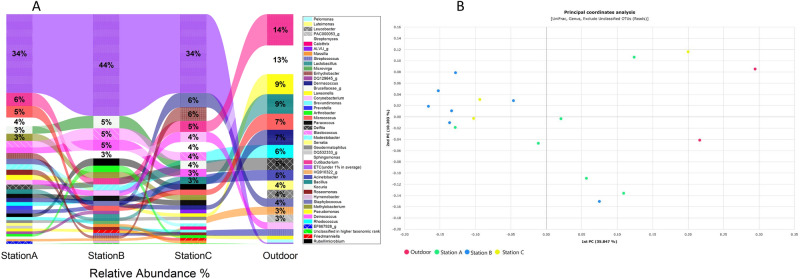
(**A**)Comparison of microbiome of SMRT stations in relation to the outdoor microbiome. Some genera including *Leucobacter* and *Luteimonas* were observed in the outdoor microbiome during this study. However, the typical skin associated *Enhydrobacter* and *Kocuria* were absent in the outdoor microbiome. (**B**) Principal Coordinate Analysis (PCoA) by UniFrac distance metric.

Microbiome and mycobiome analysis of the major taxa detected at the SMRT subway stations revealed that these taxa comprised not only normal inhabitants of skin but also of soil, plant debris and water-associated communities. The microbial composition of the major taxa present at the SMRT stations and outdoor environment are shown in Fig. 6a and b. In a previous study, it was revealed that *Acinetobacter, Sphingomonas* and *Pseudomonas* in the microbiomes of subway stations originated from outdoor sources^5^. Similarly, higher abundances of these taxa were observed in the outdoor microbiome while the microbiomes of the SMRT stations were abundant with the skin microflora (Fig. 6a, b).

There were some genera identified in both the indoor and outdoor microbiomes, such as *Bacillus, Dermacoccus, Micrococcus, Pseudomonas, Rhodococcus* and *Sphingomonas*, these taxa were more abundant in the outdoor microbiome (Table S8). An interesting observation was the very high abundances of ‘ETC’ (taxa under 1% on average) in the stations compared to those of the outdoor sample. Our results suggest that the outdoor microbiome could also have an important role in shaping the microbiomes of indoor spaces. However, some other significant and relevant factors are necessary to profile the total microbiome of an environment. For example, human activities, lifestyles and ventilation might have an extraordinary role in the making of these microbiome profiles^44^. A Comparison of β-diversity of bacterial compositions between sample types according to UniFrac distances. *(*p* < 0.05) is shown in Table 1.

**Table 1:** Comparison of β-diversity of bacterial compositions between sample types according to UniFrac distances.

	Station A	Station B	Station C	Outdoor
Station A	–	* (*p* = 0.044)	N.S. (*p* = 0.801)	* (*p* = 0.043)
Station B	N.S. (q = 0.088)	–	N.S. (*p* = 0.322)	* (*p* = 0.021)
Station C	N.S. (q = 0.801)	N.S. (q = 0.386)	–	N.S. (*p* = 0.290)
Outdoor	N.S. (q = 0.129)	N.S. (q = 0.126)	N.S. (q = 0.435)	–

The upper side showed p-value and the lower side showed q-value (adjusted *p* value).

*(*p* < 0.05).

### Chemical analysis of the aerosols at the SMRT stations

Bioaerosols consist of diverse biological and chemical sources. The intensities of microbial reactions in atmospheric aerosols may influence human health, ecosystems and atmospheric processes^45,46^. In total, 31 elements in the bioaerosols from the SMRT stations were measured in this study. The elemental compositions were similar to those of other studies, especially iron. The overall bulk aerosol iron (Fe) concentrations (average of 60.2%) were 45.4% at station B, 65.9% at station C and 69.2% at station A, similar to the results of a previous study^47^ (Table 2). Iron is the fourth-most abundant element on the earth and required by most organisms including bacteria^48^. It is also evident that iron (Fe) and selenium (Se) may have an influence in neurodegenerative diseases^49^. The Fe richness in the PMs of the SMRT stations might be influenced by the friction between the train wheels and rail lines, aerosols generated between the wheels and brake cushions and between the catenaries and the pantographs^50^. Other major elements were oxygen (average of 13%) with the highest concentration at station B (17.4%), followed by station C (11.0%) and station A (10.5%).

**Table 2:** Elemental composition of particulate matter samples collected from different subway stations obtained using WD-XRF spectrometer. (mass%).

Element	Station A	Station B	Station C	Mean	Std. dev
Al	0.9	1.49	1.01	1.13	0.31
Ba	1.59	0.89	1.62	1.37	0.41
Br	*BLD	0.03	0.01	0.01	0.02
Ca	2.47	5.28	3.22	3.66	1.46
Cl	0.23	1.58	0.6	0.8	0.7
Cr	0.13	0.16	0.13	0.14	0.02
Cu	4.44	1.62	4.37	3.48	1.61
Fe	69.2	45.4	65.9	60.17	12.89
I	0.17	*BLD	0.11	0.09	0.09
K	0.42	1.63	0.66	0.9	0.64
Mg	0.33	0.53	0.35	0.4	0.11
Mn	0.61	0.42	0.59	0.54	0.1
Mo	*BLD	0.09	*BLD	0.03	0.05
Na	0.19	0.43	0.25	0.29	0.12
Ni	0.06	0.05	0.07	0.06	0.01
Pb	0.03	0.07	0.04	0.05	0.02
Rb	*BLD	0.019	0.005	0.01	0.01
Sb	0.1	*BLD	*BLD	0.03	0.06
Sn	0.07	*BLD	0.08	0.05	0.04
Si	2.07	4.02	2.4	2.83	1.04
Sr	0.05	0.06	0.06	0.06	0.01
Te	0.13	*BLD	*BLD	0.04	0.08
Ti	*BLD	0.52	*BLD	0.17	0.3
Zn	0.44	1.18	0.59	0.74	0.39
Zr	0.36	0.24	0.34	0.31	0.06
B	0.28	0.67	0.37	0.44	0.2
C	4.39	12.9	5.14	7.48	4.71
N	0.29	2.08	0.42	0.93	1
O	10.5	17.4	11	12.97	3.85
P	0.04	0.08	0.04	0.05	0.02
S	0.49	1.19	0.62	0.77	0.37

*BDL Below detection limit.

Crustal origin elements Al, Ca, Mg, and Si were also observed at all the SMRT subway stations in this study. Interestingly, the abundance of the crustal elements was high at SMRT station B compared to the other stations. Recently, an increment in the surface particulate matter concentrations was reported in the Seoul Metropolitan area^51^.

Minor elements Ba, Cl, Cu, Mn and Zn and trace elements Br, Cr, Ti, P and Na were also present. Ba is used as one of the marker species of road dust because Ba is a chemical that is normally applied to lubricating oil to reduce smoke and engine scuff in diesel vehicles^52^. However, it is well known that Ba and Mn can be rich at subway stations^53–55^.

**Figure 7:**
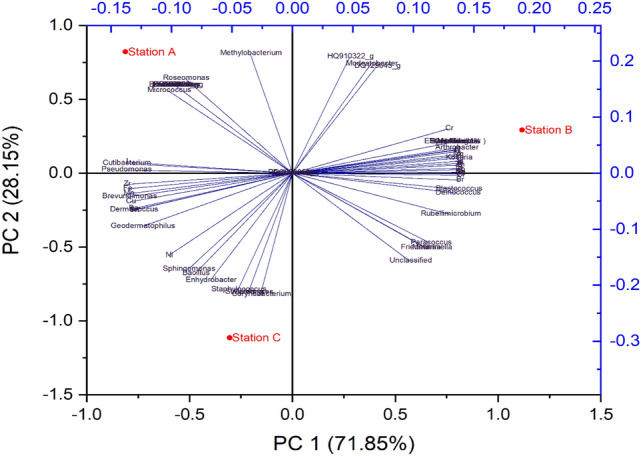
Principal Component Analysis of Chemical, bacterial and fungal components at SMRT stations.

Sodium, Potassium and Magnesium (Na, K, and Mg) are alkaline and alkaline earth elements, which are used to modify the efficacy of biodiesel production and its stability^56^, while Ca is enriched in sea spray aerosol particles^57^. The presence of these elements at the subway stations might be due to the biological activities and ambient sources of the aerosol. Due to direct releases of numerous aerosol particles (carbonaceous aerosols that include black, brown, and organic carbon) or their constituent elements such as sulfur dioxide, nitrogen oxides and volatile organic compounds, human beings have dramatically altered the structure of airborne particles^58^. One of the possible explanations for the higher abundance of human related elements such as carbon, oxygen, phosphorus and sulfur (C, O, P, and S) at station B might be the result of the large number of commuter’s activities. Human activities can enhance the amount of nitrogen (N) emitted to the aerosol, modifying the N biogeochemical cycle^59^. Our results showed increased concentrations of N at subway station B, which may involve the greater presence of commuters and their activities at the station. Principal Component Analysis in Fig. 7 shows the major components at the SMRT subway stations.

## Discussion

This is the first aerosol microbial study of SMRT subway stations, providing the bases for the further exploration of subway air quality by estimating the microbial diversity and chemical composition of aerosol particles. The current study also included the seasonal dynamics of bacterial and fungal assemblages, which were presented with the elemental composition of the particulate matter (PM) for the first time at subway stations. The most abundant bacterial phyla in the aerosols of Seoul Subway station were *Actinobacteria, Proteobacteria, Bacteriodetes, Firmicutes, Cyanobacteria, Deinococcus-Thermus* and *Choloroflexi*. The major bacterial phyla were detected, similar to the microbiomes of the aerosols at the subway stations of New York City (NYC)^12^, Hong Kong^5^, and Athens^9^. Skin-associated bacterial genera with an average abundance including *Cutibacterium* (4.7%), *Micrococcus* (3%), *Staphylococcus* (3.3%), *Enhydrobacter* (3%), *Corynebacterium* (2.7%) were detected in the aerosols of the SMRT stations. These bacterial phyla have also been detected in the aerosols of indoor building environments^60–62^ and in urban microbiomes as well^63^.

Shannon’s diversity index (H′) was larger in the SMRT subway stations (H′ _(average)_ = 4.77 ± 0.54) compared to another studies on the aerosol microbiome of Hong Kong (H′ _(average)_ = 4.13 ± 0.307)^5^ and the Barcelona subway airborne bacteria (H′ ~ 1.5)^13^. However, the SMRT bacterial diversity was less than the aerosol bacterial diversity of the Athens subway (H′_(average)_ = 5.75 ± 0.212)^9^ and the Mexico City metro (H′ _(average)_ = 6.38 ± 0.661)^4^. Overall, the airborne bacterial diversity found at SMRT stations suggests its source microbes originated mainly from human skin, oral and soil related microbiomes.

Despite having similarities with other subway stations, the SMRT showed a relatively higher diversity than those of NYC, Boston, and Barcelona, however, less than those of Athens and Mexico City. Interestingly, these microbiomes from metro systems with a high number of commuters (SMRT, Hong Kong, and NYC) and metro systems with a lower number of commuters (Boston and Oslo) share almost the same dominant bacterial genera. This result suggests that the microbial community of metro systems can be driven by human activities as most of the genera were related to the skin flora.

Overall, the microbiome of the subway stations was mostly dominated by human skin related taxa and taxa with an environmental origin (soil, water and vegetation). Within the fungal ITS libraries, the overall most abundant taxa belonged to *Ascomycota* (Mean = 56.11%) followed by *Basidiomycota* (Mean = 19.14%) similar to the findings in the Athens metro study^9^. However, the prevalence of *Basidiomycota* was significantly less at the SMRT compared to Athens (36.4%). The higher abundance of *Ascomycota* can be explained as follows: the small growth forms of *Ascomycota* are easier to aerosolize than the large growth forms of *Basidiomycota*^64^.

The iron (Fe) was the most abundant element at the subway stations including this study. A previous study on subway particles indicated that Fe-containing particles were the most common subway particle by weight concentration. Most Fe/Si-rich particles were found in the subway particle samples obtained by the portable collectors in London, accounting for 53 percent of the overall particles^65^. The iron concentrations of New York, Helsinki, Tokyo, and Budapest ranged from 40 to 92%^66–69^.

Overall, the stations included in this study harbored different phylogenetic communities based on *α- and β*-diversity comparisons. Microbial assemblages also varied depending upon the season in which the samples were taken and the station. Major elements present at the subway stations were from aerosols generated between wheels and brake cushions and between the catenaries and the pantographs. Lastly, subway stations and public transport systems generally are regarded as infection hubs for the dissemination of microbial infections and chemical influence on the public health, and our research findings provide fundamental and valuable evidence for the possible role of the public transport system in the spread of infection.

Overall, this study makes progress into the research of microbiomes and chemical components of subway station aerosols. In the future, it can be used to analyze the vulnerability of public transport and commuters to the spread of major airborne diseases in terms of potential chemical and microbial human-health hazards.

## Materials and methods

### Sample collection and processing

Particulate matter samples were collected from air purifiers installed in the platform area of the SMRT subway stations. Three stations including the most crowded one were selected for the sampling. The average daily passengers of station A, B, and C were about 76, 200 and 52 K, respectively. The sampling was done at each season during 2019 (Table 3) by recovering particulate matters collected in the medium and HEPA filters of the air purifiers operated 24 h a day for a month of period. The medium and HEPA filters were exchanged monthly, and the samples were recovered from used filters according to the maintenance schedule. The rate of air-intake was 900 m^3^/min for each air purifier. Coarse particles in the indoor air of the platform area were first removed by a pre-filter, and only fine particulate matters were collected in the medium and HEPA filters. The outdoor particulate matters in the same period of 2019 were also collected from medium filters of intake-air purification system of large buildings in the Greater Seoul Area. Each system has pre-filters and medium filters to supply clean air inside the building. The fine particle samples were recovered from the used medium filters several times in 2019 when they were exchanged. Recovered samples during the period of 2019 were merged and processed to remove coarse particles. Then, they were divided into two fractions (PM10 and > PM10) depending on their particle sizes. The separation of the two fractions was carried out using a home-made cyclonic particle separator. Samples with “19” means that the collection year was 2019.

**Table 3:** Information of SMRT stations and sampling time.

Station	Year	Month	Day	Season
Station A	2019	2	28	Winter
	2019	12	27	Winter
	2019	4	26	Spring
	2019	6	28	Summer
	2019	10	31	Autumn
	2019	11	28	Autumn
Station B	2019	2	28	Winter
	2019	12	13	Winter
	2019	4	8	Spring
	2019	6	17	Summer
	2019	6	26	Summer
	2019	8	14	Summer
	2019	9	30	Autumn
Station C	2019	2	28	Winter
	2019	4	9	Spring
	2019	6	26	Summer
PM 10	2020	3	5	–
> PM 10	2020	3	5	–

### Chemical analysis

The semi-quantitative elemental composition of the particulate matter samples collected from the three different subway stations were obtained using a ZSX Primus II WD-XRF spectrometer (Rigaku, Japan). The X-ray tube was operated in the range of 30–50 kV and 60–100 mA depending on the analyte elements. Two detectors (scintillation and gas flow counters) and eight different analyzing crystals (LiF200, PET, Ge, RX-25, RX-40, RX-45, RX-61F and RX-75) were used for effective detection of various elements. For the semi-quantitative analysis of various elements, the fundamental parameter method^70^ was applied for samples pressed into discs.

### DNA extraction and PCR amplification

Total DNA was extracted from the collected samples using the DNeasy PowerSoil Kit (Qiagen, Germany) in accordance with the manufacturer’s instructions. PCR amplification with the extracted DNA was performed using fusion primers targeting the V3 to V4 regions of the 16S rRNA gene^71,72^ and the ITS2 region^73,74^. For bacterial and fungal PCR amplification, Illumina overhang adapter primers were used (Table 4).

**Table 4:** Primers information.

Name	Sequence
16S Forward	(5ʹTCGTCGGCAGCGTCAGATGTGTATAAGAGACAG–CCTACGGGNGGCWGCAG 3ʹ)
16S Reverse	(5ʹ-GTCTCGTGGGCTCGGAGATGTGTATAAGAGACAG-GACTACHVGGGTATCTAATCC-3)
ITS3 forward	(5ʹ-AATGATACGGCGACCACCGAGATCTACAC-GCATCGATGAAGAACGCAGC-3ʹ)
ITS4 Reverse	(5ʹ-CAAGCAGAAGACGGCATACGAGAT-TCCGCTTATTGATATGC-3ʹ)

The amplifications were carried out under the following conditions: initial denaturation at 95 °C for 3 min, followed by 25 cycles of denaturation at 95 °C for 30 s, primer annealing at 55 °C for 30 s, and extension at 72 °C for 30 s, with a final elongation at 72 °C for 5 min.

The PCR products were confirmed by 1% agarose gel electrophoresis and the Chemi Doc Touch imaging system (BioRad, Hercules, CA, USA).

### Library preparation and sequencing

The sequencing libraries were prepared by a sequencing company (Chunlab, Korea). The amplified products were purified with CleanPCR (CleanNA, Netherland). Equal concentrations of purified products were pooled together, and short fragments (non-target products) were removed with CleanPCR (CleanNA, Netherland). The quality and product size were assessed on a Bioanalyzer 2100 (Agilent, Palo Alto, CA, USA) using a DNA 7500 chip (Agilent, Palo Alto, CA, USA). Mixed amplicons were pooled, and the sequencing was carried with an Illumina MiSeq Sequencing system (Illumina, USA) according to the manufacturer’s instructions.

### Data analysis

The obtained sequence reads from both the 16S rRNA genes and ITS regions were normalized to 20,000 and 10,000 reads, respectively. The normalized reads were analyzed by microbiome taxonomic profiling (MTP) of EzBioCloud (https://help.ezbiocloud.net/mtp-pipeline/)^75^. All *α-*diversity indexes and β*-*diversity of the samples were determined using MTP. At the species/phylotype and subspecies level, the EzBioCloud database has 66,303 rRNA gene sequences. Principal Coordinate Analysis (PCoA) was done with the Unifrac distance. PERMANOVA test was performed with all MTP sets including this work, it is equivalent to the “beta-group-significance” command in the QIIME2 package (https://help.ezbiocloud.net/mtp-secondary-analysis/#creating-mtp-set/).^75^ Q-values (adjusted *p* value) are obtained according to Benjamini and Hochberg^76^. Indicator value (IndVal%) analysis was done using PAST (Paleontological Statistics Software Package for Education and Data Analysis) 4.05 with default parameters^77^. Origin(Pro), Version *(2022)* OriginLab Corporation, Northampton, MA, USA, and RStudio (http://www.rstudio.com/) were used for the visualization^78^.

## Supplementary Information

Supplementary Information 1.

Supplementary Information 2.

Supplementary Information 3.

## Data Availability

The datasets generated during and/or analysed during the current study are available in the European Nucleotide Archive (ENA) repository, with the Study Accession Number PRJEB55312.

## Abbreviations

OTU: Operational Taxonomic Unit;
SMRT: Seoul Metro Rapid Transit;
PCoA: Principal Coordinate Analysis;
MTP: Microbiome taxonomic Profiling
