# Supplementary material for: Seasonal variations of the airborne microbial assemblages of the Seoul subway, South Korea from 16S and ITS gene profiles with chemical analysis
Source: Sci Rep. 2022 Nov 2;12:18456. doi: 10.1038/s41598-022-21120-8 (PMC9630434; doi:10.1038/s41598-022-21120-8)
Supplement: Supplementary file 2 — Supplementary Information 2. [file 41598_2022_21120_MOESM2_ESM.pptx]

## Slide 1
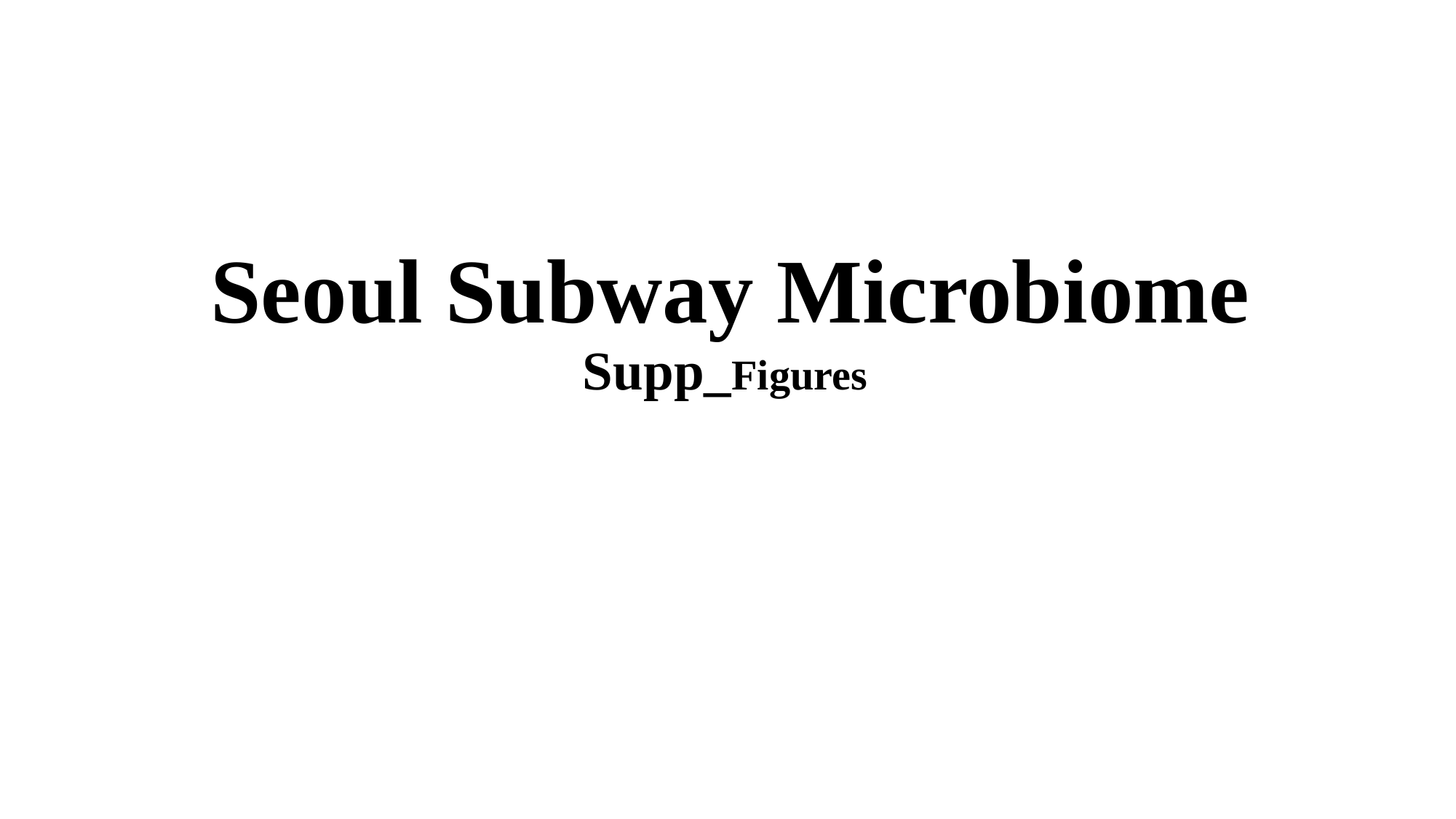

# Seoul Subway Microbiome Supp_Figures

## Slide 2
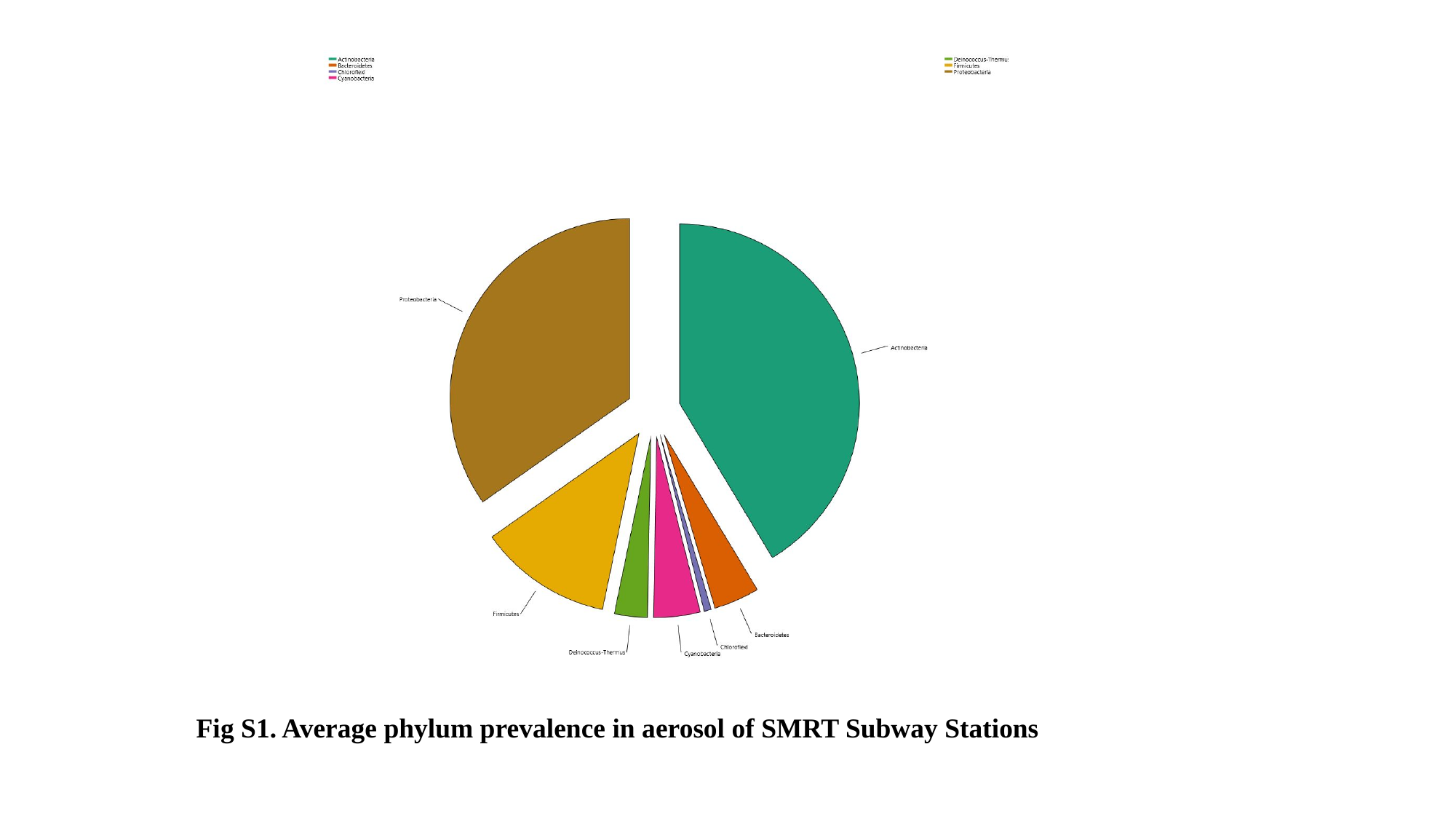

Fig S1. Average phylum prevalence in aerosol of SMRT Subway Stations

## Slide 3
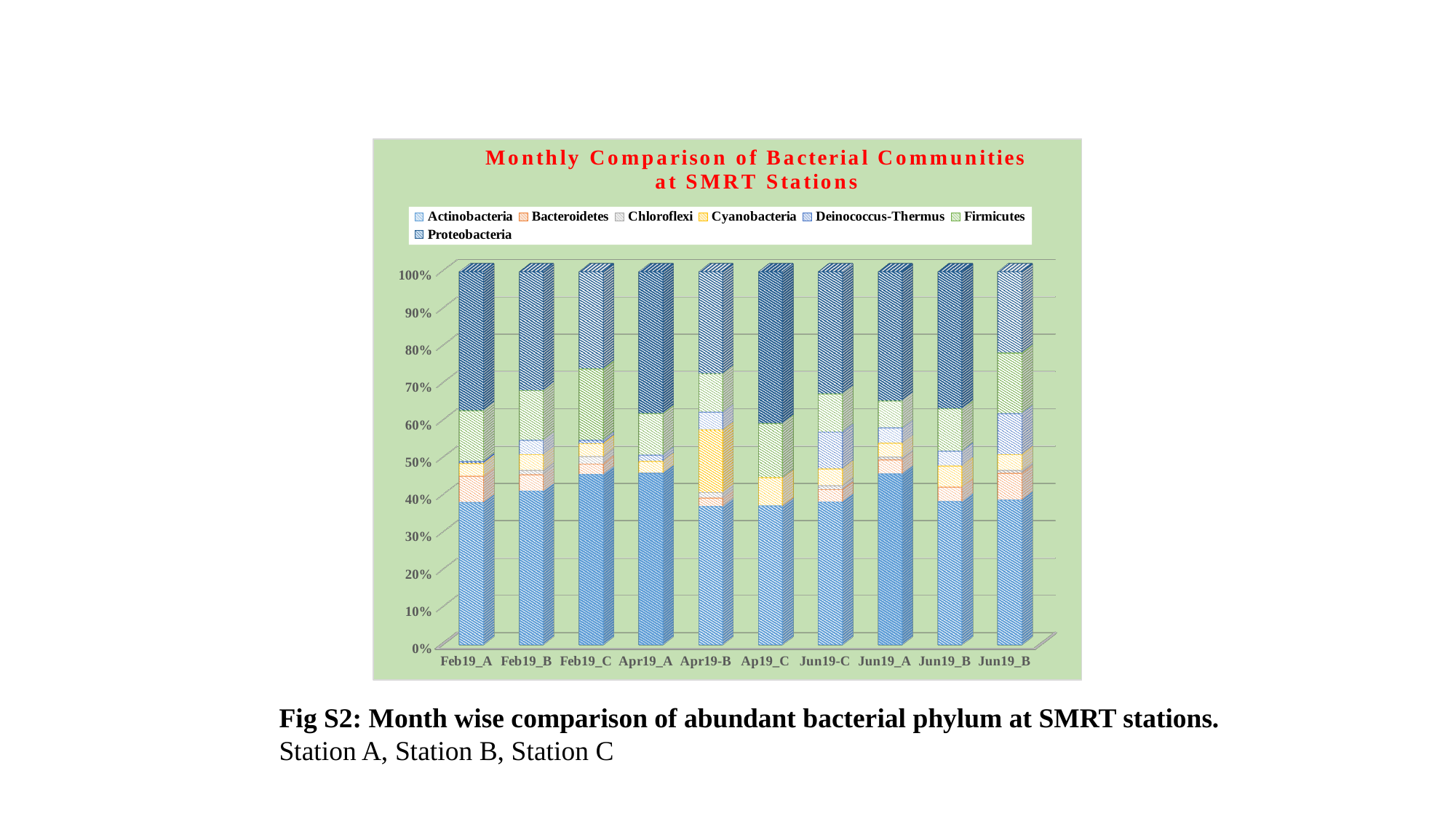

[unsupported chart]
Fig S2: Month wise comparison of abundant bacterial phylum at SMRT stations.
Station A, Station B, Station C

## Slide 4
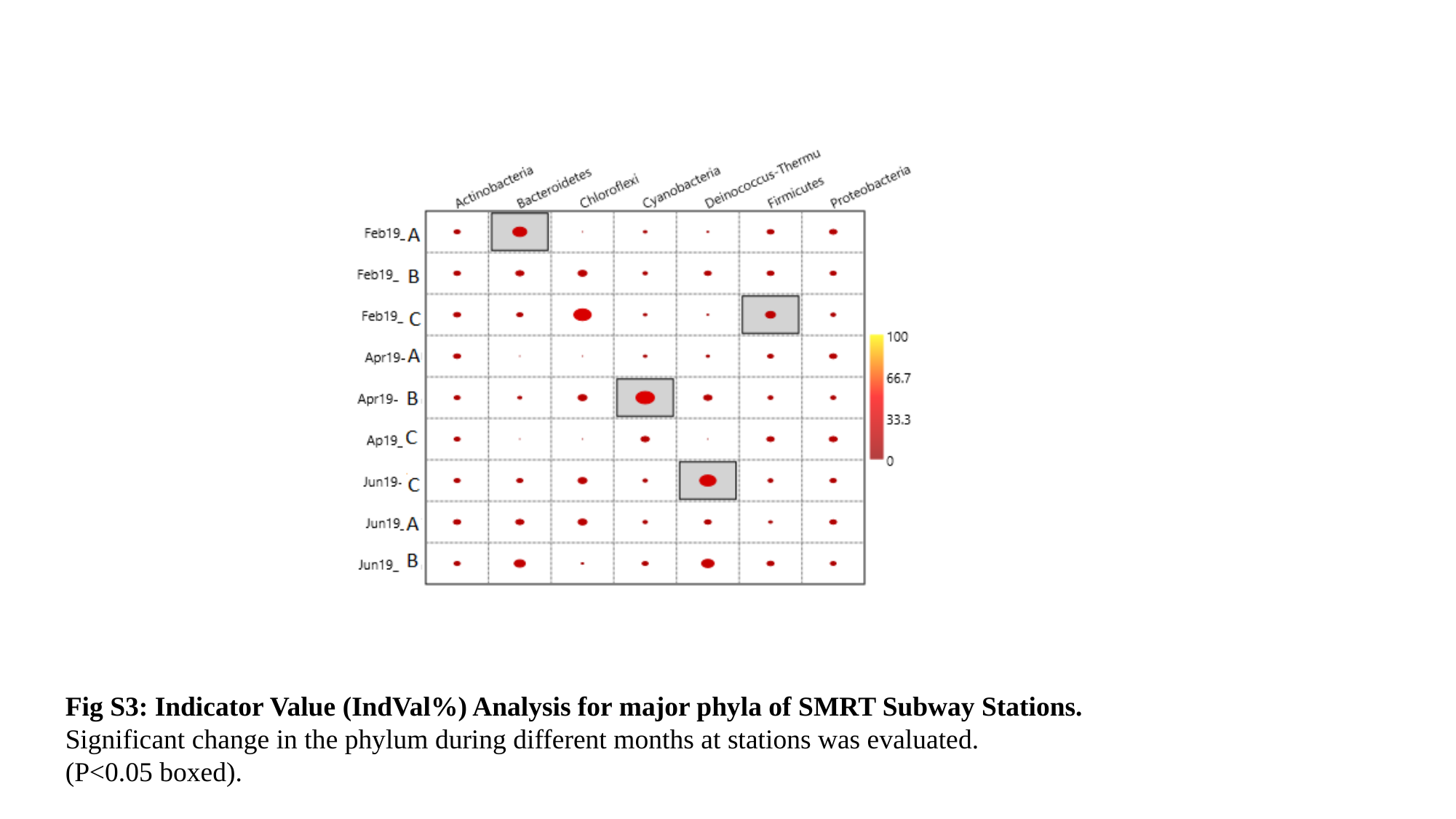

Fig S3: Indicator Value (IndVal%) Analysis for major phyla of SMRT Subway Stations.
Significant change in the phylum during different months at stations was evaluated.
(P<0.05 boxed).

## Slide 5
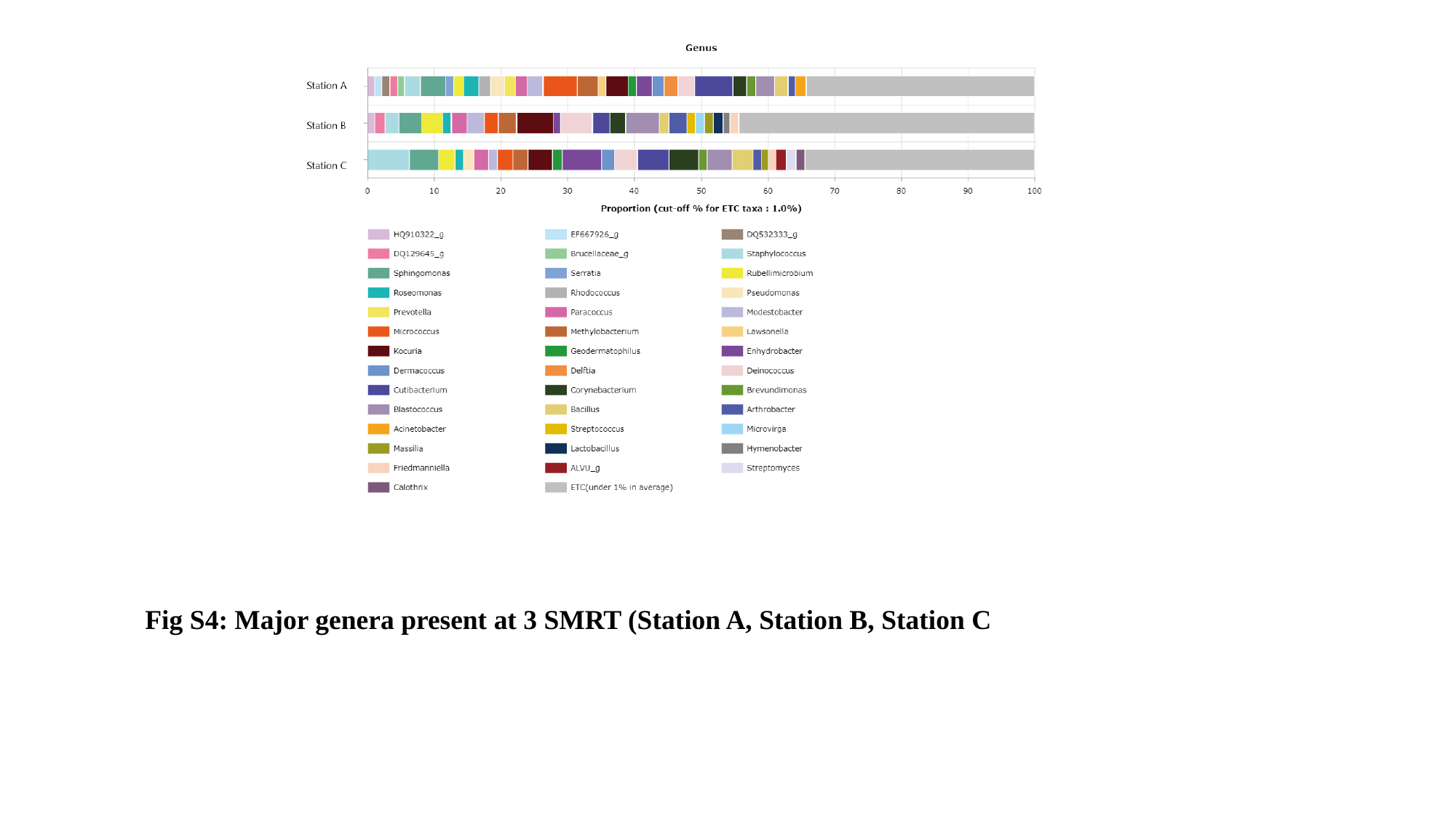

Fig S4: Major genera present at 3 SMRT (Station A, Station B, Station C

## Slide 6
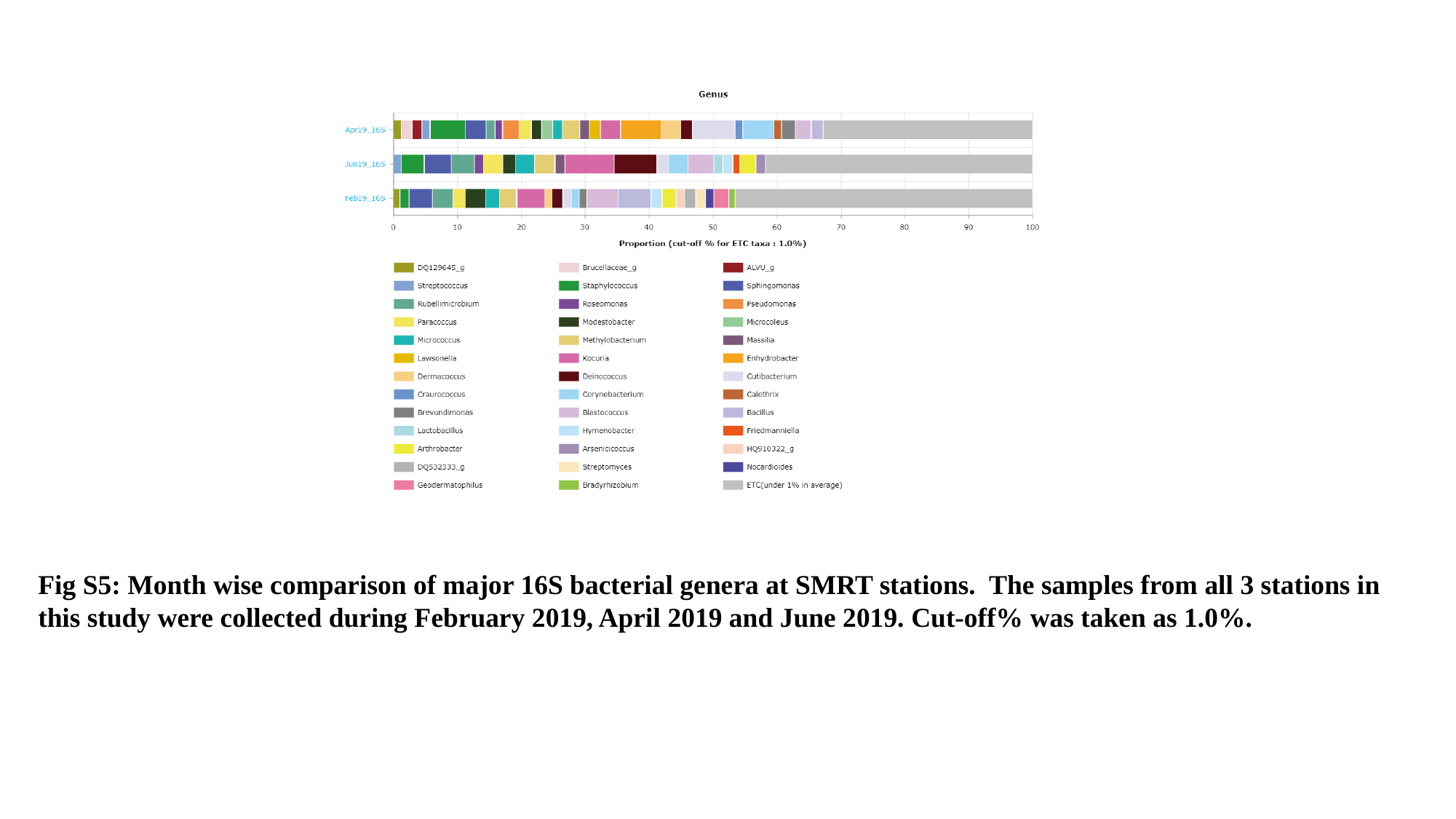

Fig S5: Month wise comparison of major 16S bacterial genera at SMRT stations. The samples from all 3 stations in this study were collected during February 2019, April 2019 and June 2019. Cut-off% was taken as 1.0%.

## Slide 7
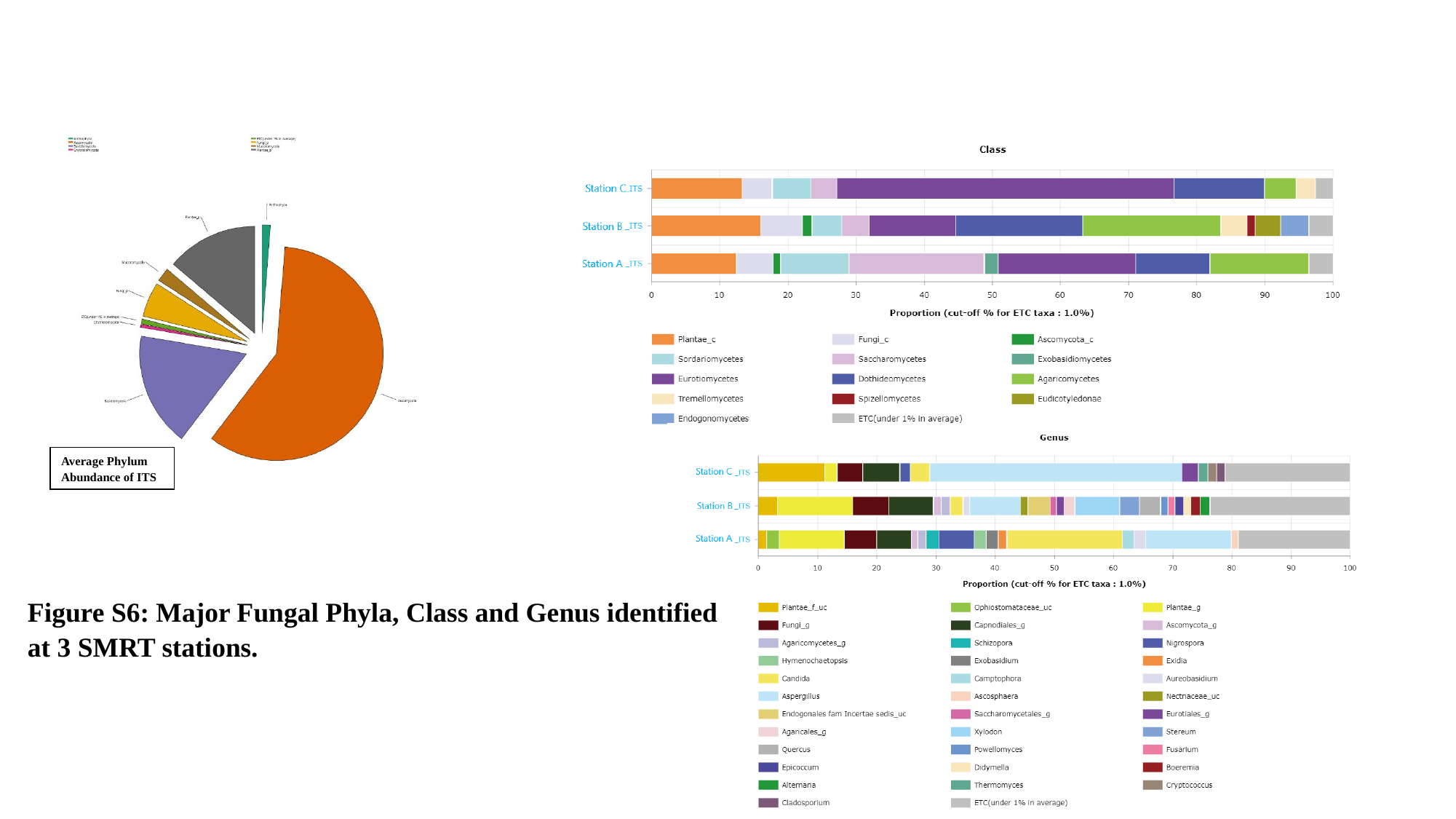

Average Phylum Abundance of ITS
Figure S6: Major Fungal Phyla, Class and Genus identified at 3 SMRT stations.

## Slide 8
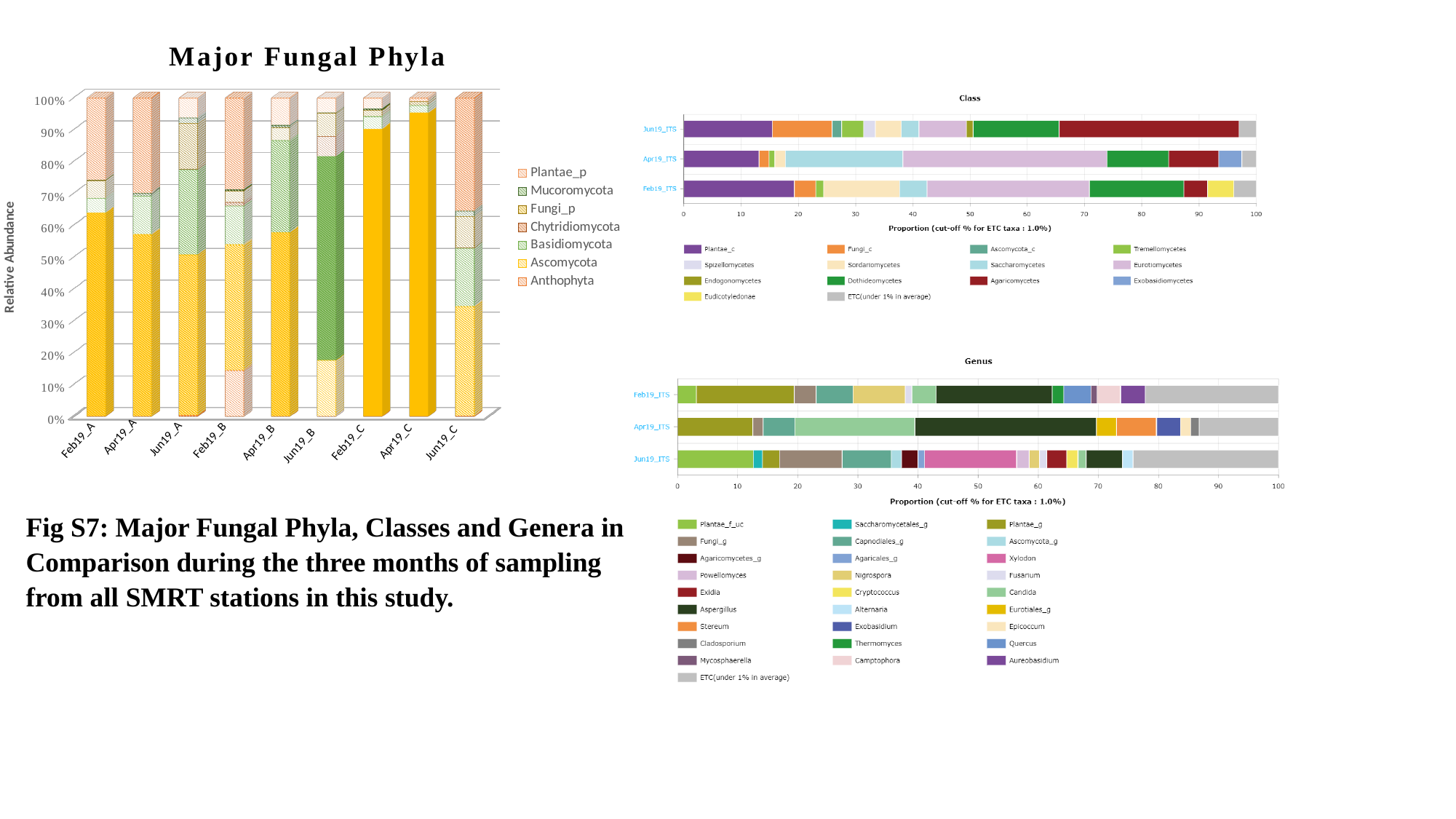

[unsupported chart]
Apr19_A
Jun19_A
Feb19_B
Feb19_A
Apr19_C
Apr19_B
Jun19_C
Feb19_C
Jun19_B
Fig S7: Major Fungal Phyla, Classes and Genera in Comparison during the three months of sampling from all SMRT stations in this study.

## Slide 9
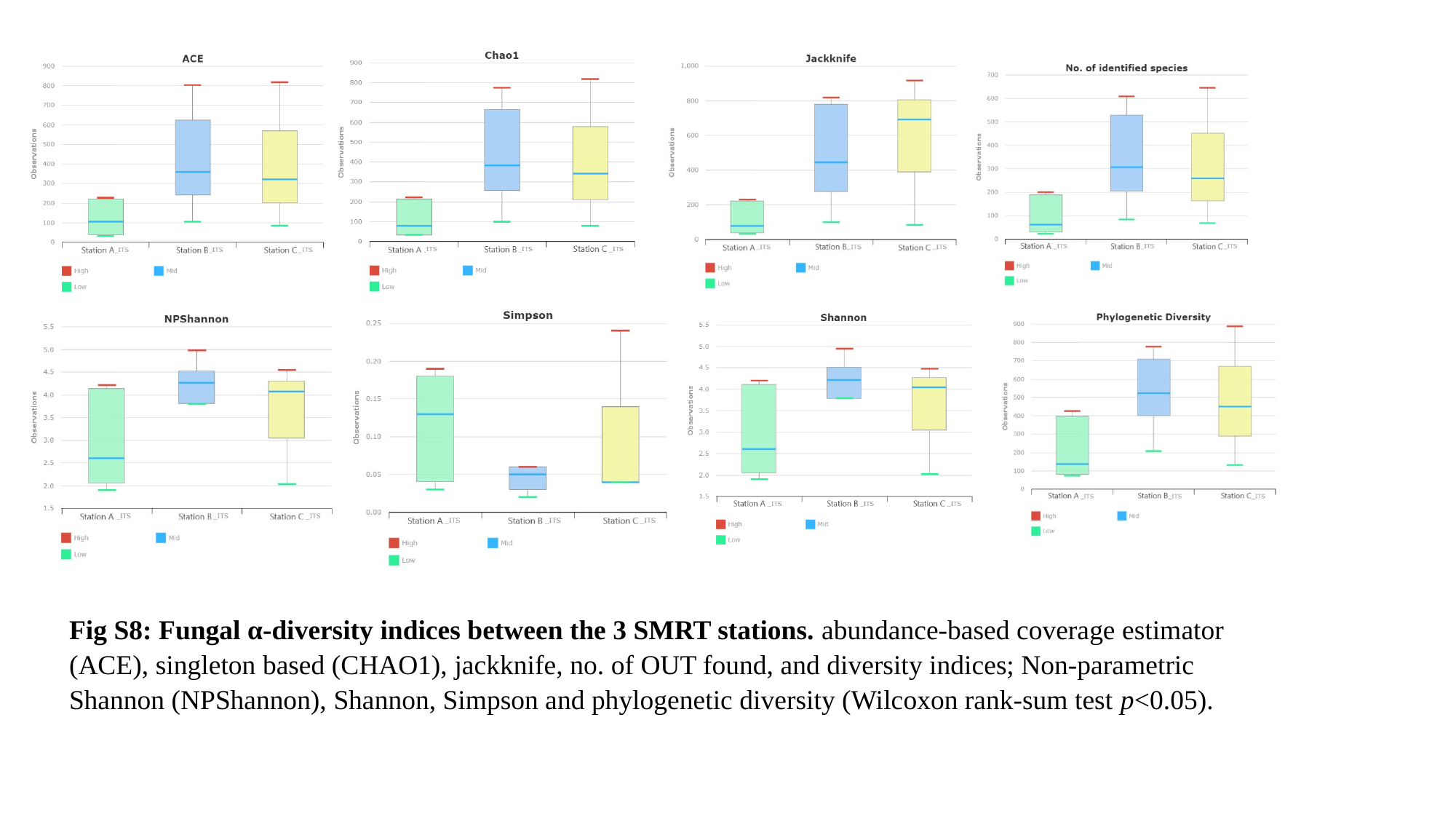

Fig S8: Fungal α-diversity indices between the 3 SMRT stations. abundance-based coverage estimator (ACE), singleton based (CHAO1), jackknife, no. of OUT found, and diversity indices; Non-parametric Shannon (NPShannon), Shannon, Simpson and phylogenetic diversity (Wilcoxon rank-sum test p<0.05).

## Slide 10
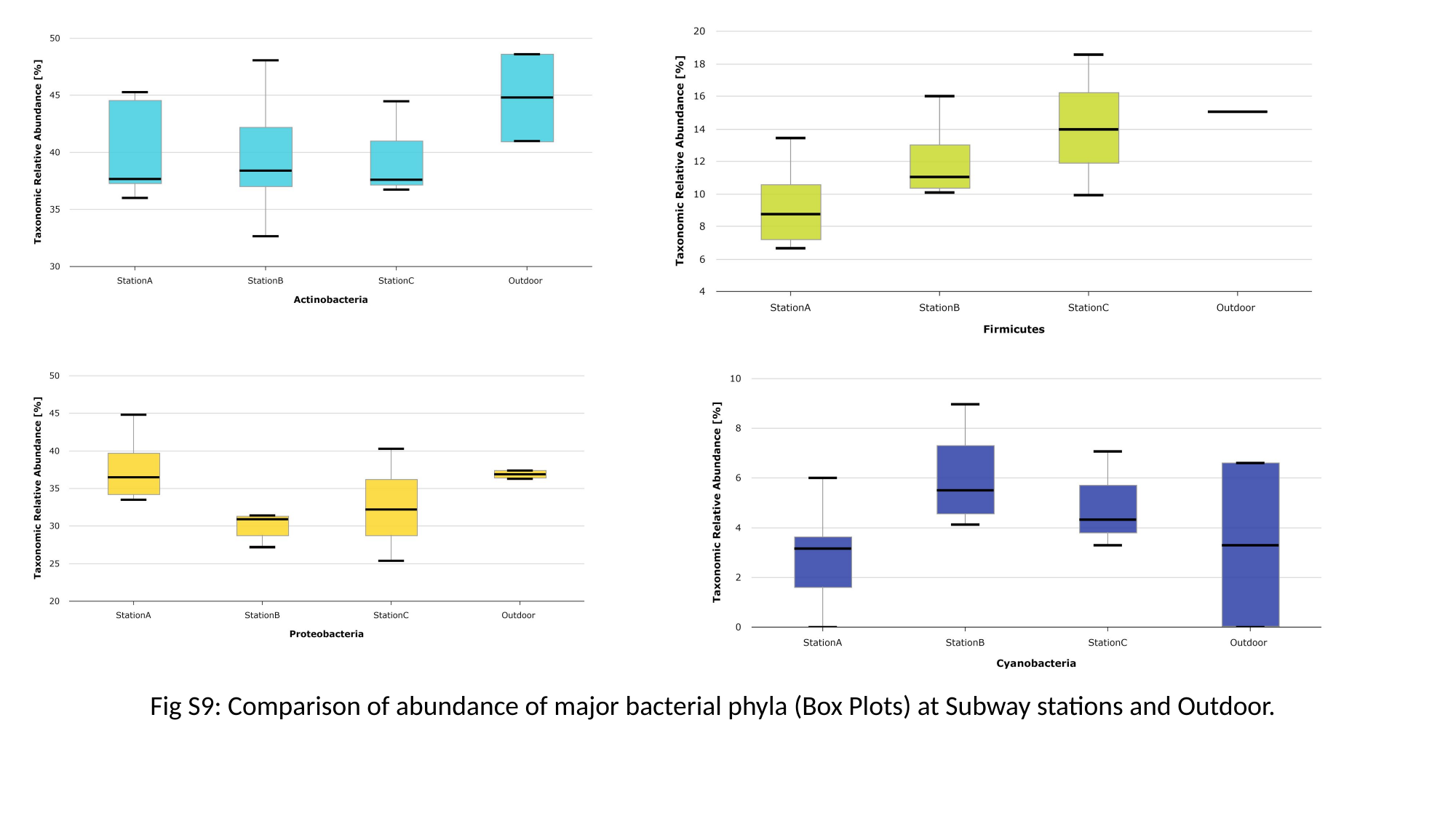

Fig S9: Comparison of abundance of major bacterial phyla (Box Plots) at Subway stations and Outdoor.

## Slide 11
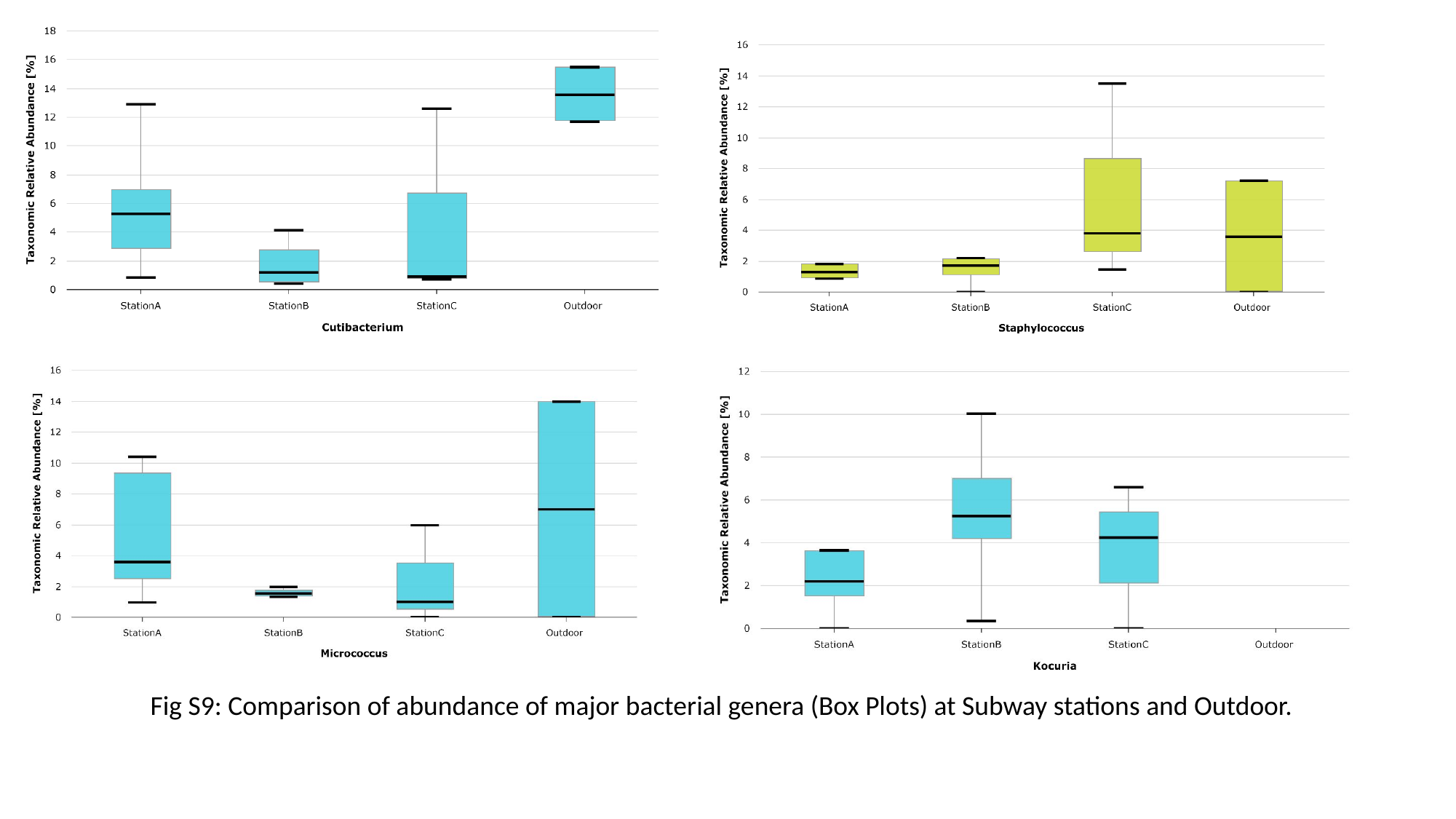

Fig S9: Comparison of abundance of major bacterial genera (Box Plots) at Subway stations and Outdoor.
