# Supplementary material for: Seasonal variations of the airborne microbial assemblages of the Seoul subway, South Korea from 16S and ITS gene profiles with chemical analysis
Source: Sci Rep. 2022 Nov 2;12:18456. doi: 10.1038/s41598-022-21120-8 (PMC9630434; doi:10.1038/s41598-022-21120-8)
Supplement: Supplementary file 3 — Supplementary Information 3. [file 41598_2022_21120_MOESM3_ESM.docx]

**Seasonal Variations of the Airborne Microbial Assemblages of the Seoul Subway, South Korea from 16S and ITS Gene Profiles with Chemical Analysis**

AUTHOR NAMES

*Zohaib Ul Hassan, Hana Cho, Changwoo Park, Yong-Hyeon Yim, Seil Kim**

AUTHOR ADDRESS

1. Group for Biometrology, Korea Research Institute of Standards and Science (KRISS), Daejeon, 34113, Republic of Korea;

2. Convergent Research Center for Emerging Virus Infection, Korea Research Institute of Chemical Technology (KRICT), Daejeon 34114, Republic of Korea;

3. Department of Bio-Analytical Science, University of Science & Technology (UST), Daejeon 34113, Republic of Korea

4. Inorganic Metrology Group, Division of Chemical and Biological Metrology, Korea Research Institute of Standards and Science, Daejeon 34113, Korea

5. Department of Agricultural Biotechnology, Seoul National University (SNU), Seoul, Republic of Korea

**MONTHLY COMPARISON OF BACTERIAL GENERA COMPOSITIONS AT SMRT STATIONS**

We compared the bacterial genera through different months of the year 2019. The bioaerosol samples were taken from all three SMRT stations during February, April and June (Fig S5). The major bacterial genera of the samples were *Kocuria* (mean abundance = 5.1%, highly rich during the June at Station with 7.6%, followed by 4.4% in February and 3.3% in April). *Kocuria* are Gram-positive, belonging to the family *Micrococcaceae*, suborder *Micrococcineae*, order *Actinomycetales* ^1^, broadly scattered into natural environments and can also be found often as normal flora of skin and oral cavity of humans and mammals ^2^. Furthermore, the average abundance of *Bacillus* was 2.4%, with a peak during February (5.2%), 1.9% in April and not detected during June, contrasted to the study ^3^ suggested the lower percentage of *Bacillus* during winter (Supplementary Table S3).

**MONTHLY COMPARISON OF FUNGAL COMPOSITION AT SMRT SUBWAY STATIONS**

We compared the fungal communities the three different months for the SMRT stations included in this study. The samples were collected during February, April and June from all stations included in this study. *Ascomycota* was identified as the most abundant phylum (mean abundance) during February and April comprising of (64.5%) and (69.7%), followed by *Basidiomycota* (6.8%) and (14.2%) respectively. Whereas, *Basidiomycota* was most abundant (36.3%) during June, indicating the potential impact of high temperatures and wind in summer. *Ascomycota* levels were significantly lower than in previously mentioned months. However, plant related genera were remain consistent throughout these months. Unclassified Fungal phylum was an outlier, with a noteworthy rise in June. In addition to this, *Chytridiomycota* and *Mucoromycota* were a prevalence during June and absent in other months. Considering the stations, we got some attention-grabbing outcomes, *Ascomycota* was substantially predominant at SMRT station C during the month of February and April with (>90%) of the relative abundance, however, the abundance of *Ascomycota* were significantly reduced in June (reduced up to 34.3%). Paralleled trends at other two stations represented the low temperature enrichment of *Ascomycota* during February and April and sunk in high temperatures. Contrastingly, *Basidiomycota* substantively higher in hot temperature during June at all SMRT stations in this study Fig S7. The analysis at class level revealed that *Eurotiomycetes* revealed as a major fungal class in above mentioned months (Mean=24.1%), peaked at subway station C in April and February. Compare to this, the abundance appeared to be on lower side at other stations throughout the time frame of the sampling. Some other interesting findings were the abundances of *Agariomycetes* (Mean=14.8%), highest in June at all the stations. On the other hand, *Dothideomycetes* (Mean=10.5%) revealed greater occurrence in February and June, almost negligible during April. Major contribution to profusion of *Saccharomycetes* evaluated from station A during April. Due to the lack of studies on month wise comparison of airborne fungi in subway systems, we could not compare directly these results with others studies. However, class level analysis results of our study have some resemblance with the outcomes obtained in swine houses in South Korea ^4^. *Aspergillus* was the dominant genus (mean= 18.5%), with a peak richness in April (30.2%), February (19.3%) and in June (6.0%). *Candida* (Mean=8.4%) most abundant in April (20%) similar to the *Aspergillus*. Other major genera were Unclassified *Capnodiales*_g (Mean=6.49%), *Xylodon* (Mean=5.10%) highly found during June Fig S7.

1. Stackebrandt, E., Koch, C., Gvozdiak, O. & Schumann, P. Taxonomic dissection of the genus Micrococcus: Kocuria gen. nov., Nesterenkonia gen. nov., Kytococcus gen. nov., Dermacoccus gen. nov., and Micrococcus cohn 1872 gen. emend. *Int. J. Syst. Bacteriol.* **45**, 682–692 (1995).

2. Savini, V. *et al.* Drug sensitivity and clinical impact of members of the genus Kocuria. *Journal of Medical Microbiology* **59**, 1395–1402 (2010).

3. Fang, Z. *et al.* Profile and Characteristics of Culturable Airborne Bacteria in Hangzhou, Southeast of China. *Aerosol Air Qual. Res.* **16**, 1690–1700 (2016).

4. Kumari, P., Woo, C., Yamamoto, N. & Choi, H. L. Variations in abundance, diversity and community composition of airborne fungi in swine houses across seasons. *Sci. Rep.* **6**, 1–11 (2016).
